# Sexuality of Disabled Athletes Depending on the Form of Locomotion

**DOI:** 10.1515/hukin-2015-0094

**Published:** 2015-01-12

**Authors:** Ryszard Plinta, Joanna Sobiecka, Agnieszka Drosdzol-Cop, Agnieszka Nowak-Brzezińska, Violetta Skrzypulec-Plinta

**Affiliations:** 1 School of Health Sciences in Katowice, Medical University of Silesia, Katowice, Department of Adapted Physical Activity and Sport, Chair of Physiotherapy, Katowice, Poland; 2 Faculty of Motor Rehabilitation, University School of Physical Education, Krakow, Poland; 3 School of Health Sciences in Katowice, Medical University of Silesia, Katowice, Chair of Woman’s Health, Katowice, Poland; 4 Institute of Computer Science, Faculty of Computer Science and Material Science, Silesian University, Sosnowiec, Poland

**Keywords:** Paralympic games, paralympians, physical activity, sexual functioning

## Abstract

The main purpose of this study was to determine sexuality of disabled athletes depending on the form of locomotion. The study included 170 disabled athletes, aged between 18 and 45. The entire population was divided into 3 research groups depending on the form of locomotion: moving on wheelchairs (n=52), on crutches (n=29) and unaided (n=89). The research tool was a questionnaire voluntarily and anonymously completed by the respondents of the research groups. The questionnaire was composed of a general part concerning the socio-demographic conditions, medical history, health problems, a part dedicated to physical disability as well as the Polish version of the International Index of Erectile Function (IIEF) and the Female Sexual Function Index (FSFI) evaluating sexual life. STATISTICA 10.0 for Windows was used in the statistical analysis. Subjects moving on crutches were significantly older than ones moving on wheelchairs and unaided (34.41 ±11.00 vs. 30.49 ±10.44 and 27.99 ±10.51 years, respectively) (p=0.018). Clinically significant erectile dysfunctions were most often diagnosed in athletes moving on wheelchairs (70.27%), followed by athletes moving on crutches and moving unaided (60% and 35.42%, respectively; p=0.048). Clinical sexual dysfunctions were diagnosed on a similar level among all female athletes. It was concluded that the form of locomotion may determine sexuality of disabled men. Males on wheelchair revealed the worst sexual functioning. Female athletes moving on wheelchairs, on crutches and moving unaided were comparable in the aspect of their sexual life.

## Introduction

The Polish athletes first competed at the 1972 Summer Paralympic Games in Heidelberg, Germany. At present, the Polish paralympic team is composed of 94 competitors (60 men and 34 women). The participation in the Paralympic Games is a reward for years of immense effort and hard work. The athletes rely not only on their natural predispositions and talent, but must undergo specific training, which is not substantially different from that of fully abled athletes. It is a great challenge that carries significant health implications (Silver, 2004; Sobiecka et al., 2008).

Physical disability may affect physical functioning, a mood, the quality of life (QoL) and restrict sexual and non-sexual contacts. Yet, the data on the QoL and sexuality of physically disabled men and women is scarce, especially with regard to the correlations between physical activity and the above-mentioned variables (Alexander et al., 2009; Lombardi et al., 2010; Silver, 2004; Sobiecka et al., 2008).

Numerous studies have demonstrated that professional physical rehabilitation may significantly improve the QoL in men and women after spinal cord injury, which emphasizes the role of physical activity in people with physical disabilities (Alexander et al., 2009; Lombardi et al., 2010). Considering that the data on disability (in particular spinal cord injury) emphasizes the beneficial impact of regular physical activity on both physical and psychological health, and knowing that very few studies have explored the beneficial effect of physical activity on sexual functions, sexual satisfaction and sexual well-being, exploring the relationship between various levels of physical activity, sexual function and satisfaction in individuals with disability may constitute an innovative and interesting research area (Alexander et al., 2009; Lombardi et al., 2010; Silver, 2004; Sobiecka et al., 2008;).

The main purpose of this study was to determine the quality of sexual functioning of disabled athletes depending on the form of locomotion. This is the first study focusing on the sexuality of Polish athletes with disabilities.

## Material and Methods

### Participants

The study encompassed 170 Polish athletes with physical disabilities, aged between 18 and 45. The entire population was divided into 3 research groups depending on the form of locomotion: moving on wheelchairs (n=52), on crutches (n=29) and unaided (n=89).

The disabled athletes were recruited from Polish sport clubs for individuals with disabilities. The inclusion criteria for the study population were as follows: the membership of Polish sport clubs for people with disabilities, regular physical activity, consent to participation in the study, complete filling out of the questionnaire, general good health and age range between 18 and 45 years old.

In fact, 67 individuals had a spinal cord injury (39.41%), the rest constituted athletes with different inherited or acquired disease (e.g. bone/muscle inherited diseases, phocomelia, states after surgical or accidental limb amputation).

### Procedures

For the final analysis only healthy subjects were qualified; individuals with any comorbidities (e.g. diabetes mellitus, hypertension, any endocrinological diseases) were excluded at the beginning of the research. The exclusion criteria for every study group were as follows: lack of consent to participation in the study, incomplete filling out of the questionnaire and age range under 18 or over 45 years old.

The research program was approved by the Bioethics Committee of the Medical University of Silesia in Katowice, Poland.

### Measures

The research tool was a questionnaire voluntarily and anonymously completed by the respondents of the research and control groups. The questionnaire was composed of a general part concerning the socio-demographic conditions (age, marital status, education, occupational activity, physical activity), medical history, health problems, a part dedicated to physical disability (the reason of disability, diagnosis, form of locomotion) and a detailed part in the form of self-evaluation inventories: the Polish version of the International Index of Erectile Function (IIEF) and the Female Sexual Function Index (FSFI) evaluating male and female sexual functioning.

### International Index of Erectile Function (IIEF)

The IIEF is a multidimensional, 5-grade instrument for self-evaluation of all male sexual functions within the previous 4 weeks. It is characterised by high validity, reliability, sensitivity and test-retest reliability in the diagnosing of changes, confirmed by over 50 clinical trials. Implementation of the IIEF is a recommended standard in the diagnosis and evaluation of erectile dysfunctions and their intensification (Cappelleri et al., 1999; Lue et al., 2004; Rosen et al., 2002).

The IIEF questionnaire encompasses 15 items grouped in 5 collective domains (subscales) describing: I - erectile function, II - orgasm function, III - sexual desire, IV - intercourse satisfaction and V - overall satisfaction. The total scores within all the domains (I–V) create positive dependence with correct sexual functioning. An additional analysis of the erectile subscale facilitates the isolation of four disorder intensification levels (Erectile Dysfunction - ED): erectile function (26–30 points), mild ED (17–25 points), moderate ED (11–16 points) and severe ED (6–10 points). Clinically significant erectile dysfunction is diagnosed at values equal to or less than 25 points (cut-off point) (Cappelleri et al., 1999; Lue et al., 2004; Rosen et al., 2002).

### Female Sexual Function Index (FSFI)

The FSFI has been confirmed and clinically documented with regard to validity, sensitivity, reliability, internal consistency, stability and test-retest reliability in diagnosing disorders of sexual desire, arousal, orgasm as well as dyspareunia (Ferenidou et al., 2008; Meston, 2003; Rosen et al., 2000).

The FSFI is composed of 19 items divided into 6 collective domains (subscales): I – sexual desire, II – sexual arousal, III - lubrication, IV - orgasm, V – sexual satisfaction and VI – dyspareunia. The final results are obtained separately for each of the subscales by summing up the elementary points encompassed within each of the 6 domains and a selected coefficient. The interpretation of partial results is linear dependence: the higher the score, the better the sexual functioning within a given category. The next stage is a global evaluation of the entire FSFI scale. Results below 65% of the maximum number of points scored in each of the domains (less than 3.9 points) were considered as sexual dysfunction in that domain. In a global FSFI assessment, clinically significant female sexual disorders (FSD) were diagnosed at values lower or equal to 26.55 points. Sexual disorders were diagnosed according to the American Psychiatric Association’s Diagnostic and Statistical Manual of Mental Disorders Fourth Edition (DSM-IV) and the American Foundation for Urologic Disease (AFUD) criteria (scores of 26.55 or less on the FSFI and 3.9 or less in each of its domains, with the presence of sexual distress) (Ferenidou et al., 2008; Meston, 2003; Rosen et al., 2000).

### Statistical analysis

For the data analysis, STATISTICA 10.0 for Windows was used. Differences among parameters were considered significant at the level of 0.05. The statistical analysis included the Shapiro-Wilk test, the Mann-Whitney U-test, the Pearson’s chi*-*square test, Kruskal-Wallis covariance and post-hoc tests.

## Results

### Participants – socio-demographic characteristics

All participants (100%) were Polish, White/Caucasian. The research groups were comparable with regard to the place of residence, marital status, education and occupational activity. Subjects moving on crutches were significantly older than ones moving on wheelchairs and unaided (34.41 ±11.00 vs. 30.49 ±10.44 and 27.99 ±10.51 years, respectively) (p=0.018) (Table 1).

Generally, the research groups did not differ statistically with regard to the age at the first intercourse, length of the current relationship, frequency of sexual intercourse and the number of sexual partners (Table 2).

### Sexual functioning of men with disabilities – IIEF scores

The holistic evaluation of the IIEF scale and its five collective domains showed statistically significant differences only in the erectile function (p=0.048), as well as the orgasm function (p=0.046) between the groups. Men moving unaided showed the best sexual functioning (Kruskal-Wallis covariance test) (Table 3).

Clinically significant erectile dysfunctions were most often diagnosed in athletes moving on wheelchairs (70.27%) with approximately a 40% result of mild erectile dysfunctions, followed by athletes moving on crutches and moving unaided (results of 60% and 35.42% respectively; p=0.048) (Table 3).

### Sexual functioning of women with disabilities – FSFI scores

The holistic evaluation of the FSFI scale showed no statistically significant differences in the FSFI global score and its six collective domains. Female athletes moving on wheelchairs, on crutches and moving unaided were comparable in the aspect of sexual functioning (the Kruskal-Wallis covariance test) (Table 4).

Implementing the cut-off points, statistically significant clinical sexual dysfunctions concerning desire, arousal, lubrication, orgasm, satisfaction, pain as well as the global FSD were diagnosed at the similar level among all female athletes (Table 4).

**Figure 1:**
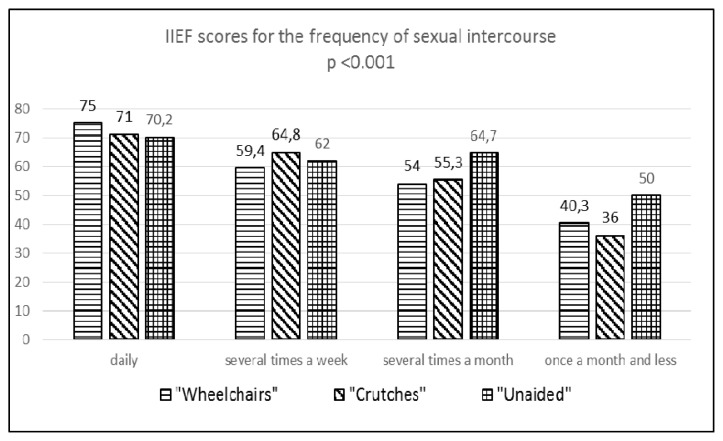
IIEF scores for the frequency of sexual intercourse in the studied men IIEF - International Index of Erectile Function

An additional statistical analysis showed that the frequency of sexual intercourse statistically positively correlated with the IIEF global score (p<0.001) (Figure 1). These differences with FSFI scores were not statistically significant.

## Discussion

There are numerous studies on the QoL, psychological aspects and sexuality of people with disabilities after spinal cord injury. Some studies have focused on the effect of physical activity in the form of rehabilitation on general well-being (Consortium for Spinal Cord Medicine, 2010; Dinomais et al., 2010; Tasiemski and Brewer, 2011; Vall et al., 2011). However, there is a paucity of data regarding groups of athletes with disabilities who practice sports. Our study is one of the first on the subject, and offers unique findings on this important issue. We showed the comparison of sexuality of disabled athletes depending on the form of locomotion. It was found that the form of locomotion might determine sexuality of disabled men as males on wheelchair revealed the worst sexual functioning. Female athletes moving on wheelchairs, on crutches and unaided were comparable in the aspect of sexual functioning.

Medical publications report that people with spinal cord injury demonstrate a series of disabilities and limitations (including general physical health, the QoL, psychological functioning, social and personal relations, as well as sex life) (Alexander et al., 2009; Kreuter et al., 2011; Reitz et al., 2004; Vall et al., 2011).

These results are consistent with our first group – athletes moving on wheelchairs. However, human sexuality is a complex and multidimensional phenomenon and many additional factors, including culture, a social context, age, mental health, and interpersonal relations, may influence the sexual function of men and women. Therefore, the interpretation of our results should be careful. It was difficult to isolate additional factors affecting personal sexuality among study participants.

Spinal cord injuries can have a significant impact on sexual functioning. The majority of clinical papers indicate that spinal cord injury affects particularly men’s sexual behaviour and that the sex life of women with spinal cord injury remains less affected than among men (Alexander et al., 2009; Kreuter et al., 2011; Reitz et al., 2004; Vall et al., 2011). Male athletes moving on wheelchairs are frequently after spinal cord injury which affects to a large extent their sexual function. The main reasons for sexual disorders are neurological disturbances after spinal cord injury.

In our results the percentage of FSD did not differ significantly between the study groups. By contrast, clinically significant ED were diagnosed quite frequently (athletes moving on wheelchairs - 70.27%, on crutches - 60%, moving unaided - 35.42%). It is well-known that female sexuality is mostly depended on mental well-being and interpersonal relations.

Numerous studies demonstrate that professional medical help, especially physical rehabilitation, might significantly improve physical and mental well-being, as well as sexual function in men and women after spinal cord injury, which emphasizes the special role of physical activity in the management of physically disabled people (Alexander et al., 2009; Lombardi et al., 2010; Silver, 2004).

The Consortium for Spinal Cord Medicine published the clinical practice guideline for health-care professionals on sexuality and reproductive health in adults with spinal cord injury with a view to encourage individuals to take an active role in obtaining information related to sexual issues, as well as encourage people with spinal cord injury to explore the role of sexuality in their lives and various ways in which they may express their sexuality. The professionals suggest developing a sexual education and treatment plan with the individual consistent with the results of the sexual history, an interview, a relationship status and physical exam findings (Consortium for Spinal Cord Medicine, 2010).

Currently, there are only two articles in the PubMed database evaluating general wellbeing and the QoL of athletes with disabilities. However, they did not include any information about sexual functioning. Tasiemski and Brewer (2011) in their study, examined the interrelationships among athletic identity, sport participation and psychological adjustment in a sample of people with spinal cord injury. The authors concluded that being able to practice one’s favourite sport after injury was associated with higher levels of athletic identity and better psychological adjustment. Team sport participants reported experiencing better psychological adjustment than individual sport participants (Tasiemski and Brewer, 2011).

Dinomais et al. (2010) investigated social functioning, the quality of life and self-esteem in 496 young athletes with disabilities taking part in adapted competitive sports. The researchers noticed significantly higher social functioning scores in this population, which confirms the positive effect of sport on the general well-being of physically disabled people (Dinomais et al., 2010).

The design of our study offers important insights into the understanding of the association between the form of locomotion and sexual functioning of Polish athletes with disabilities. Firstly, by including a numerous group of Polish athletes with disabilities, we endeavoured to ensure reliable results. Secondly, the use of self-reporting in evaluating sexual functioning encouraged the participants to express openly the majority of their problems. Thirdly, this is one of the first clinical research evaluating sex life in such a specific group of athletes.

Despite all these advantages, the limitations of the study should be recognized. Firstly, the study sample may be too small to generalize the obtained results to the entire population of Polish people with physical disabilities. Secondly, individuals who were particularly uncomfortable talking about their sex/intimate life may have been less likely to respond truthfully. Thirdly, we did not access the detailed information about spinal cord injury levels and severity (e.g. ASIA scores, medical history). The data was based only on questionnaires responses. Finally, the authors did not concentrate on non-intercourse sexual activity (e.g. fellatio, cunnilingus), which is described as a major component of routine sexual activity among individuals with disabilities. This fact might affect the results of the present study. Moreover, the authors did not isolate additional factors effecting personal sexuality among study participants. Therefore, the interpretation of results concerning sexuality should be careful.

The form of locomotion may determine sexuality of disabled men. Males on wheelchair revealed the worst sexual functioning. Female athletes moving on wheelchairs, on crutches and moving unaided were comparable in the aspect of sexual functioning.

## Figures and Tables

**Table 1:** Socio-demographic characteristics of the study population (mean±SD/n(%))

Variables		The form of locomotion			p

		Wheelchairs	Crutches	Unaided	
Age (years)		30.49±10.44	34.41±11.00	27.99±10.51	p=0.018
Residence	Rural areas	9(17.31%)	5(17.24%)	26(29.55%)	NS (p=0.19985)
	Town <100,000	17(32.69%)	14(48.28%)	27(30.68%)	
	Big city >100,000	26(50.00%)	10(34.48%)	35(39.77%)	
Marital status	Single	39(75.00%)	20(71.43%)	68(78.16%)	NS (p=0.75103)
	Married	13(25.00%)	8(28.57%)	19(21.84%)	
Education	Primary	1(1.88%)	1(3.45%)	6(6.74%)	NS (p=0.63642)
	Vocational	10(18.87%)	3(10.34%)	17(19.10%)	
	Secondary	28(52.83%)	18(62.07%)	50(56.18%)	
	Tertiary	14(26.42%)	7(24.14%)	16(17.98%)	
Occupational activity	Unemployed	13(25.00%)	7(24.14%)	19(21.35%)	NS (p=0.57907)
	Employed	26(50.00%)	16(55.17%)	39(43.82%)	
	Student	13(25.00%)	6(20.69%)	31(34.83%)	
Cause of physical disability	Inherited	16(69.81%)	11(37.93%)	48(54.55%)	p=0.01425
	Spinal cord injury	31(58.49%)	10(34.48%)	26(29.55%)	p=0.00253
	Disease	7(13.21%)	8(27.59%)	12(13.79%)	NS (p=0.17175)

SD – standard deviation; NS – not significant

**Table 2:** Sexual behaviours in the study population (mean±SD; %)

Variables		Wheelchairs	Crutches	Unaided	Kruskal-Wallis test
Age at the first intercourse (years)		18.47±4.38	19.91±5.65	18.86±3.34	NS (p=0.420531)
Length of current relationship (years)		8.28±9.71	7.03±6.75	7.34±7.18	NS (p=0.852457)
The number of sexual partners		6.98±9.63	5.95±6.71	6.17±9.61	NS (p=0.880026)
The frequency of sexual intercourse n(%)	once a day	1(2.43%)	1(5.00%)	6(10.00%)	NS (p=0.68896)
	several times/week	13(31.71%)	4(20.00%)	19(31.67%)	
	several times/month	15(36.59%)	8(40.00%)	21(35.00%)	
	1 or less/month	12(29.27%)	7(35.00%)	14(23.33%)	

SD – standard deviation; NS – not significant

**Table 3:** IIEF scores in studied men (mean±SD; min-max; %)

IIEF domains		Wheelchairs	Crutches	Unaided	Kruskal- Wallis test
IIEF global score	Mean ± SD	50.80±18.17	49.83±19.69	55.20±19.60	NS (p=0.1543)
	Min-Max	8–75	4–71	5–75	
Erectile function	Mean ± SD	19.80±8.46	20.47±8.47	22.57±9.14	0.048
	Min-Max	1–30	1–30	0–30	
Orgasm function	Mean ± SD	6.58±3.20	7.47±3.24	7.96±3.04	0.046
	Min-Max	1–10	1–10	1–10	
Sexual desire	Mean ± SD	7.93±1.99	7.89±1.57	8.36±1.87	NS (p=0.2369)
	Min-Max	2–10	4–10	2–10	
Intercourse satisfaction	Mean ± SD	9.20±4.36	9.18±4.03	9.19±4.70	NS (p=0.8205)
	Min-Max	0–15	0–15	0–15	
Overall satisfaction	Mean ± SD	7.30±2.23	7.88±2.15	7.74±2.50	NS (p=0.3011)
	Min-Max	2–10	4–10	2–10	
Erectile Dysfunction	No ED n(%)	11(29.73%)	6(40.00%)	31(64.58%)	0.048
	Mild ED n(%)	16(43.24%)	8(53.33%)	12(25.00%)	
	Moderate ED n(%)	6(16.22%)	1(6.67%)	4(8.34%)	
	Severe ED n(%)	4(10.81%)	0(0.00%)	1(2.08%)	

SD – standard deviation; NS – not significant; IIEF - International Index of Erectile Function; ED - Erectile Dysfunction

**Table 4:** FSFI scores and sexual dysfunctions in studied women (mean±SD; min-max; %)

	FSFI domains	Wheelchairs	Crutches	Unaided	Kruskal-Wallis test
FSFI global score	Mean ± SD	21.34±11.56	21.70±10.35	22.10±11.77	NS (p=0.8936)
	Min-Max	4.40–34.2	3.6–34.5	2–34.5	
Desire	Mean ± SD	3.60±1.20	2.91±1.57	3.78±1.18	NS (p=0.3692)
	Min-Max	1.2–5.4	0–4.8	1.2–6	
Arousal	Mean ± SD	3.4±2.65	3.13±2.28	3.39±2.37	NS (p=0.7631)
	Min-Max	0–6	0–5.7	0–6	
Lubrication	Mean ± SD	3.1±2.65	3.6±2.52	3.9±2.63	NS (p=0.4196)
	Min-Max	0–6	0–6	0–6	
Orgasm	Mean ± SD	3.11±2.66	3.43±2.45	3.67±2.30	NS (p=0.9071)
	Min-Max	0–6	0–6	0–6	
Satisfaction	Mean ± SD	3.91±1.90	3.83±1.92	4.50±1.66	NS (p=0.3394)
	Min-Max	0–6	0–6	0.8–6	
Pain	Mean ± SD	4.22±2.51	4.29±2.16	3.43±2.8	NS (p=0.8789)
	Min-Max	0–6	0–6	0–6	
Female sexual dysfunctions n(%)	Desire disorders	7(70.00%)	3(33.33%)	16(72.73%)	NS (p=0.10437)
	Arousal disorders	4(40%)	4(44.44%)	11(50%)	NS (p=0.86365)
	Lubrication disorders	3(30.00%)	4(44.44%)	9(40.91%)	NS (p=0.78419)
	Orgasmic disorders	3(30.00%)	4(44.44%)	10(45.45%)	NS (p=0.69811)
	Satisfaction disorders	3(30.00%)	3(42.86%)	5(23.81%)	NS (p=0.62707)
	Pain disorders	2(20.00%)	4(44.44%)	7(31.82%)	NS (p=0.52012)
	Global FSD	4(40.00%)	2(28.57%)	14(56.00%)	NS (p=0.37627)

SD – standard deviation; NS – not significant; FSFI – Female Sexual Functioning Index
